# Integrated enhanced Raman scattering: a review

**DOI:** 10.1186/s40580-021-00290-7

**Published:** 2021-12-03

**Authors:** Sahand Eslami, Stefano Palomba

**Affiliations:** 1 Center for Nano Science and Technology (CNST), Istituto Italiano di Tecnologia, Via Morego 30, 16163 Genoa, Italy; 2 Institute of Photonics and Optical Science (IPOS), The University of Sydney, Camperdown, NSW 2006 Australia; 3 The University of Sydney Nano Institute, The University of Sydney, Camperdown, NSW 2006 Australia

**Keywords:** Enhanced Raman scattering, Molecular sensor, Nanophotonic sensor, Plasmonics, Optical nose, Artificial nose

## Abstract

The demand for effective, real-time environmental monitoring and for customized point-of-care (PoC) health, requires the ability to detect low molecular concentrations, using portable, reliable and cost-effective devices. However, traditional techniques often require time consuming, highly technical and laborious sample preparations, as well as expensive, slow and bulky instrumentation that needs to be supervised by laboratory technicians. Consequently, fast, compact, self-sufficient, reusable and cost-effective lab-on-a-chip (LOC) devices, which can perform all the required tasks and can then upload the data to portable devices, would revolutionize any mobile sensing application by bringing the testing device to the field or to the patient. Integrated enhanced Raman scattering devices are the most promising platform to accomplish this vision and to become the basic architecture for future universal molecular sensors and hence an artificial optical nose. Here we are reviewing the latest theoretical and experimental work along this direction.

## Introduction

The capability of detecting individual molecules without the a priori knowledge of the molecule to detect is the Holy Grail of molecular sensing. Detecting and identifying single molecules has a plethora of applications that spans from pollution to biomarkers for disease, DNA sequencing, odor detection, forensic science, airborne pathogen identification, molecular composition of gas and liquids, etc. The closest sensor of this kind we know is, for instance, the human olfactory system. This is highly discriminative, being able to identify up to 10^9^ volatile compounds [1], by just using 400 olfactory receptors [2]. Furthermore, this leads to sensing and categorizing an uncountable number of odors, which are just a combination of specific individual compounds. Although a single receptor interacts with multiple different molecules without an a priori knowledge [3], the olfactory system can be defined as “label free” and therefore universal. The universal identification and detection of molecular species would serve well the point-of-care (POC) diagnostics, which is gaining attention especially if integrated with micro/nanofluidic systems. These would underpin the fundamental infrastructure of future self-sufficient lab-on-a-chip (LOC) platforms [4]. POC is supposed to enhance the modern trend toward personalized medicine offering a faster, cost-effective and remote analytical outcome which fits well into the current virtual medicine framework. However, current molecular sensors are not fully label-free and are not fully integrated in a LOC platform. Label-free sensors exhibits huge benefits not only because they do not need any prior preparation based on the a priori knowledge of the molecule to detect but also because of flexibility and reusability: a true label-free molecular sensor could become the universal standard that serves many sectors detecting and identifying molecules of interest in both volatile and liquid environment. In order to reach this Holy Grail, if even possible, it is essential to develop sensors capable of detecting and identifying individual molecules. A possible universal phenomenon capable of delivering the molecular “fingerprints” is Raman scattering [5].

Hence, future universal molecular sensors are envisioned to be fully integrated in mobile smartphones where they can detect biomolecules and biomarkers for specific diseases, perform blood test analysis, “sniff” drugs or alcohol in breath, or measure the presence of pollutants in the water or air. Such devices need to be small, reusable and able to collect, manipulate and deliver the fluid (liquid or gas) under test to the sensor.

Raman spectroscopy (RS) appears to fit for purpose and it is considered to be an ideal method, as it can provide highly resolved molecular vibrational information [6, 7]. Although RS can detect a molecule’s unique chemical signature, the Raman light scattered by a single molecule is a very inefficient process. Compared to fluorescence cross sections a Raman cross section could be estimated to be roughly a million times lower, in case of the most favorable resonant Raman scattering conditions [8]. Hence, it is paramount to amplify Raman signals in order to make it viable as a potential future universal sensing platform. It is well-known that surface-enhanced Raman spectroscopy (SERS) [9] is an effective label-free analytical platform which is capable of detecting the unique molecular fingerprints of various molecules in various media (gas or liquid) [5]. SERS requires plasmonic, i.e. metallic, nanostructures to achieve its full potentials. Indeed, metal surfaces create the condition to enhance the local electromagnetic fields generated by the coupling of light at resonance with the free electron plasma, i.e. the plasmonic resonance (PR). Plasmonic nanostructures are known to enormously enhance Raman scattering [10]. This enhancement factor (EF) is particularly high when the molecule is in very close proximity (nanometer scale) to the light confined in the gap between two metallic particles or metal films, forming a metal-dielectric-metal (MDM) system. A more realistic estimation of the EF in these conditions brings it to be up to a maximum of 10^10^, thanks to the field “hot spot” that could be generated in the interstice between nanometer scale plasmonic dimers.

Since the first fortuitous discovery of SERS [11, 12] there have been tens of thousands articles published about it and for sure there’s no lack also of good reviews [13–15]. We will here discuss and review a selected approach to SERS based on plasmonic waveguide geometries, which in our view could be very well suited for the future design of possible integrated molecular LOC sensors for compact and commercially viable on-chip devices.

## Raman scattering theory

Among the various sensing platforms, optics-based devices have shown substantial progress and promise, especially those utilizing plasmonic nanostructures [16]. However, in most cases, metallic nanostructures need to be functionalized in order to capture and immobilize the targeted molecule before detection, hence requiring labelling and consequently an a priori knowledge of the molecule of interest. Moreover, the light is scattered in all directions requiring complicated and often bulky optics to efficiently collect the photons. Waveguide-based sensors are an ideal platform when the scattered photons are coupled to the waveguide mode rather than be emitted in free-space.

On-chip devices that guide and manipulate light require nanophotonic structures that can confine light, such as dielectric-based (photonic) waveguides. However, these are limited by diffraction and cannot compress light tighter than $\lambda /n$, where $\lambda$ is the wavelength of light and $n$ is the refractive index of the dielectric.

It is well-known that when light is coupled to the free electrons of a metal film at the interface with a dielectric, a coherent oscillation of electrons results from the light-matter interactions in particular phase matching conditions. Here, a propagating surface wave, called Surface Plasmon Polariton (SPP), is generated. This surface wave maintains the optical frequency of the exciting light exhibiting a smaller effective wavelength and larger wavevector than the one in vacuum (${k}_{0}$), since it exhibits evanescent characteristics. This mechanism gives rise to the peculiar subwavelength light confinement and enhancement [17–19].

When two metal-dielectric interfaces, supporting SPPs, are brought in close proximity, e.g. < 1000 nm for the telecom wavelength and < 450 nm in the visible [20], forming an MDM structure, then the two SPPs modes interact with each other generating a subwavelength confined mode, called “gap-plasmon mode”, which is supported at any wavelength, exhibiting no cut-off, hence confining the field indefinitively regardless the gap size [20, 21]. The propagation constant of the gap-plasmon mode, ${k}_{MDM}$, and its effective mode index, ${n}_{MDM}={\mathrm{Re}[k}_{MDM}]/{k}_{0}$, strongly depend on the gap size of the MDM structure. The smaller is the gap, the higher ${n}_{MDM}$ and stronger attractive Coulomb forces across the gap increase the charge accumulation, enhancing the near-field intensity. For instance, with a gap size of $\sim 3 \, \text{nm}$, ${n}_{MDM} \sim 7$ at ${\lambda }_{0}=600\,\text{ nm}$, so that the wavelength of the gap-mode is more than 7 times smaller than in air, i.e. ${\lambda }_{MDM}={\lambda }_{0}/{n}_{MDM}\cong$ 86 nm. At these dimensions, both the phase velocity and the group velocity of the mode are strongly reduced, leading to high field enhancement. The key characteristic of the gap-plasmon mode is that the smaller the gap, the stronger the field confinement. This is because the group velocity (${\upsilon }_{g}$) becomes infinitively small the larger the propagation constant ${dk}_{MDM}$, since ${dk}_{MDM}/d\omega =1/{\upsilon }_{g}$ where $\omega$ is the angular frequency. However, the larger the field confinement, the shorter the propagation length due to a deeper penetration of the field into the metal, which consequently increases the losses [22–24]. This unprecedented mode confinement enormously enhances light-matter interactions such as fluorescence [25–27], nonlinear optical properties [28–30] and Raman scattering [31–34].

The ultimate goal in chemical analysis is not only the sensitivity at the single molecule level but also the possibility to identify the type of molecule without any labelling protocol and therefore any a priori knowledge of the molecule to detect. Raman spectroscopy can not only detect single molecules but can also provide their unique chemical signature. However, the Raman signal scattered by a single molecule is very weak, with a typical cross-section of ~ 10^–25^ cm^2^/molecule [35]. For comparison, fluorescent molecules have cross-sections of approximately 10^−16^ cm^2^/molecule [36, 37]. In 1977 it was observed that the Raman signal drastically increases when the molecules are located on a rough silver surface—SERS [11, 38]. Such enhancement is due to the localized surface plasmon resonance that metal particle aggregates exhibit in the nearfield [39, 40]. Generally, the linear Raman scattering intensity of free molecules depends on the dipole generated by the molecule, ${\mathbf{p}}_{m}\left({\omega }_{R}, {\mathbf{r}}_{m}\right)={{\varvec{\upalpha}}}_{m}\left({\omega }_{0},{\omega }_{R}\right){\mathrm{G}}_{1}\left({\omega }_{0},{\mathbf{r}}_{m}\right){\mathbf{E}}_{0}\left({\omega }_{0}\right),$ where ${{\varvec{\upalpha}}}_{m}\left({\omega }_{0},{\omega }_{R}\right)$ is the polarizability, ${\mathbf{E}}_{0}\left({\omega }_{0}\right)$ is the incident field strength and ${\mathrm{G}}_{1}\left({\omega }_{0},{\mathbf{r}}_{m}\right)$ is the electromagnetic EF of the incident field at the molecule’s position ${\mathbf{r}}_{m}$. If an optical antenna, which can supply ${\mathrm{G}}_{1}\left({\omega }_{0},{\mathbf{r}}_{m}\right)$ > 1, is placed at a position ${\mathbf{r}}_{A}$ near the molecule, then the antenna itself is locally excited by the nearby molecule. Consequently, the induced dipole is added to the dipole generated by the molecule, both contributing to the signal measured by a detector in the far field [41, 42]. Therefore, the total Raman intensity can be expressed by

1 $$I\left({\omega }_{R}\right)\propto {\left|{\mathrm{G}}_{1}\left({\omega }_{0},{\mathbf{r}}_{m}\right)\right|}^{2}{\cdot \left|{\mathrm{G}}_{2}\left({\omega }_{R},{\mathbf{r}}_{A}\right){\alpha }_{m}\left({\omega }_{0},{\omega }_{R}\right){\mathbf{E}}_{0}\left({\omega }_{0}\right)\right|}^{2}$$

where ${\mathrm{G}}_{2}\left({\omega }_{R},{\mathbf{r}}_{A}\right)$ is the EF generated by the induced dipole at the antenna location [43, 44]. This shows that the total enhanced Raman intensity depends on two factors, one is the local field enhancement ${\mathrm{G}}_{1}\left({\omega }_{0},{\mathbf{r}}_{m}\right)$ and the other is the apparent Raman polarizability ${\mathrm{G}}_{2}\left({\omega }_{R},{\mathbf{r}}_{A}\right){\alpha }_{m}\left({\omega }_{0},{\omega }_{R}\right)$, which is due to the mutual excitation between the molecule and the antenna [45]. Typically, noble metal nanostructures can efficiently act as both a receiving and transmitting antenna at optical frequencies with strong local electromagnetic field, hence giant Raman scattering enhancement. Since the incident and the Raman scattered frequencies are usually comparable, and thus ${\mathrm{G}}_{1}\approx {\mathrm{G}}_{2}$, the total plasmonic enhancement is approximately proportional to the fourth power of the EF,

2 $$I\left({\omega }_{R}\right)\propto {\left|\mathrm{G}\right|}^{4}{\sigma }_{m}{I}_{0}\left({\omega }_{0}\right)$$

where ${\sigma }_{m}\propto {\left|{\alpha }_{m}\right|}^{2}$ is the molecule scattering cross-section in free-space and $\mathrm{G}={\mathrm{G}}_{1}\cong {\mathrm{G}}_{2}$ is the EF [41, 46, 47].

The EF of a plasmonic dimer consisting of two metal nanostructures separated by a gap, i.e. an MDM nanostructure, increases enormously as the gap becomes smaller, e.g. for a gold nanoparticle dimer with gap size from $10$ to $2\, \text{nm}$, the ${\mathrm{G}}_{\mathrm{TOT}}={\left|\mathrm{G}\right|}^{4}$ increases from ${10}^{5}$ to ${10}^{9}$ [45]. Generally, for a good plasmonic metal like silver, $\mathrm{G}\ge$ 10^2^ which implies that ${\mathrm{G}}_{\mathrm{TOT}}\ge$ 10^8^ [48]. Such enhancement brings the Raman cross section to be comparable to the fluorescence one. Moreover, a molecule can go through more Raman cycles than fluorescence cycles due to the shorter vibrational relaxation time compared that for the electronic level. Therefore, a molecule can scatter approximately ${10}^{2}{-}{10}^{3}$ more Raman photons in one lifetime cycle of a fluorescence process. Nevertheless, fluorescence in average produces still more photons than Raman scattering due to the much higher cross-section [49].

Modern state-of-the-art SERS-based sensors combine universal identification and single molecule detection of Enhanced Raman Scattering (ERS) with microfluidic systems. However, the presence of the microfluidic environment increases the noise generated by the limited SERS-active centers. This can be mitigated by enlarging the detection volume and the number of analyte molecules bonded to SERS-active centers [50]. Nevertheless, all these platforms still need to bond the targeted molecule to the SERS-active center, which therefore is not fully label-free. Combining microfluidic systems with integrated photonics/plasmonics/hybrid architecture to detect single molecules in a truly label-free platform via ERS is still at its birth [51, 52].

## Theoretical formalism for waveguide-assisted Raman scattering

**Fig. 1:**
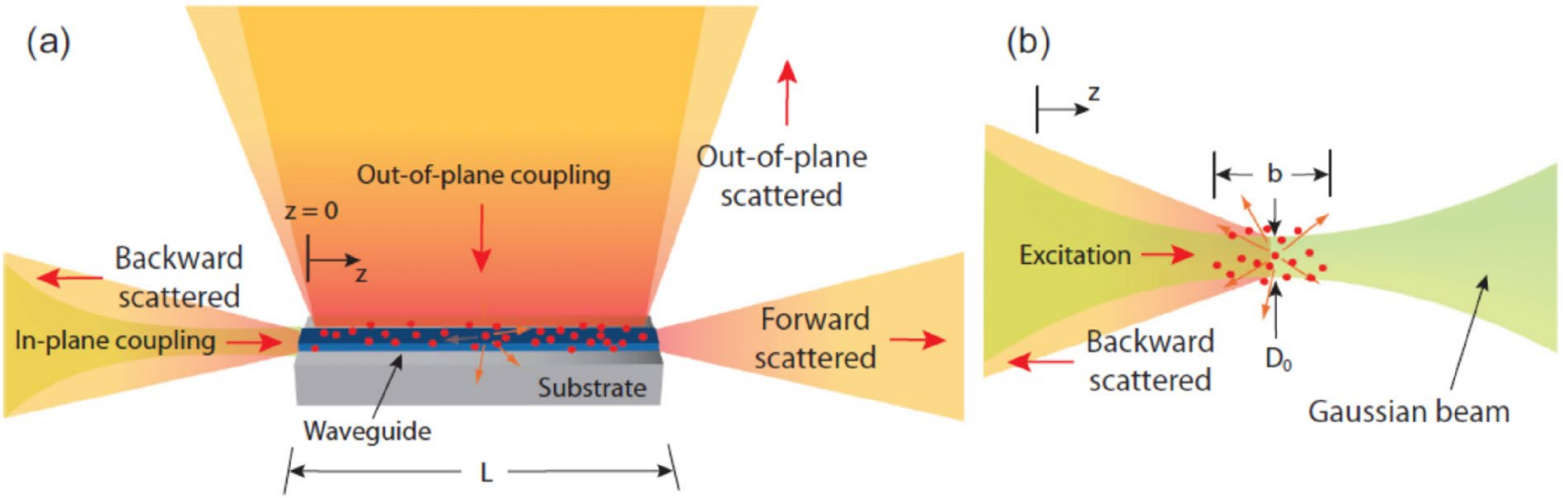
**a** Schematic of different configurations of laser excitation and Raman scattered light collection with respect to the WG of length L oriented in the light guiding z direction. Excitation is either (i) in plane via coupling to the WG mode or (ii) out-of-plane from the top. Collection is either in the (I) forward, (II) backward, or (III) out-of-plane scattered direction. **b** Schematic of laser excitation and Raman scattered light collection in free space. Excitation is in the forward direction and Raman scattered light is collected in the backward direction. The Gaussian beam is propagating in the z direction with waist diameter D_0_ and depth of focus b. (Reprinted with permission from Ref. [53])

In ‘classic’ Raman scattering, the Raman emission is usually collected in the backward direction to exclude the background of excitation light (Fig. 1b). A Raman active molecule in proximity of a waveguide can be excited either by in-plane coupling, i.e. the waveguide mode, or by out-of-plane coupling (Fig. 1a).In either case, the Raman emission can be detected both by out of plane scattering and by the light coupled back into the waveguide and collected from one or the other end. In this way a waveguide-based platform adds the flexibility not only because the signal can be forward or backward collected, but also because it could augment the collection efficiency by potentially collect the signal by both sides at the same time. Moreover, this platform can decouple the excitation form the collection, i.e. out-of-plane excitation.

**Fig. 2:**
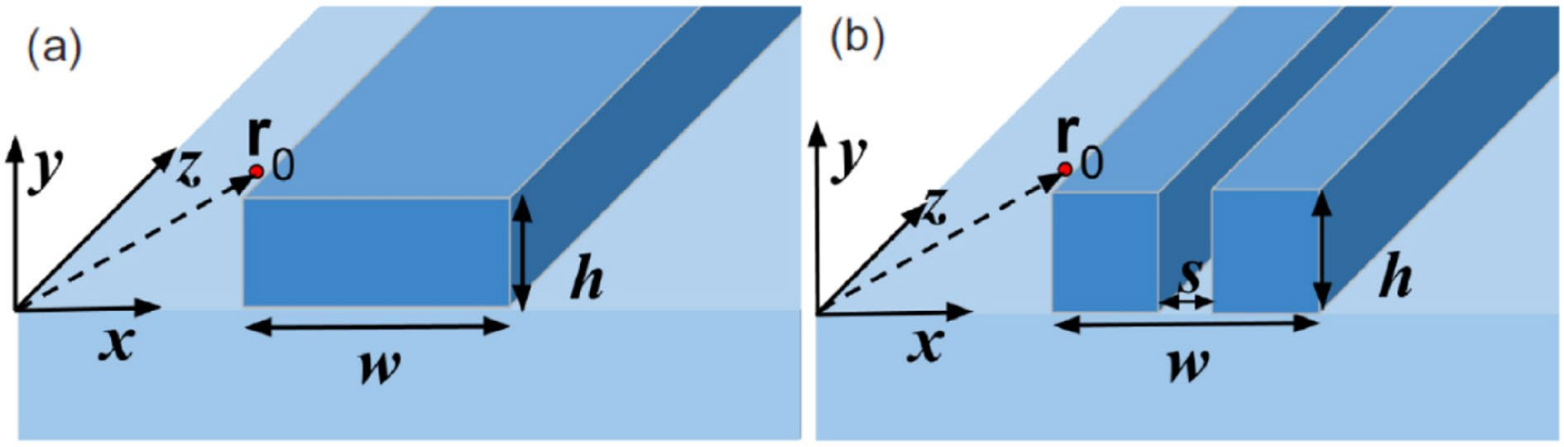
Schematic of a dielectric **a** strip and **b** slot waveguides defined by a core of higher refractive index (Si_3_N_4_, TiO_2_ or silicon) patterned on a lower index bottom cladding (typically silicon dioxide). The scattering particles are assumed to be embedded with a uniform density in the upper cladding (colorless region). A particle at position **r**_0_ is shown for both cases. (Reprinted with permission from Ref. [54])

Coupling light into waveguide mode has few advantages such as maintaining a compact design, a stable and rugged sensing device, a signal collection by the same waveguide, a compatibility with mature Silicon/Si_3_N_4_-technology that allows integration, multiplexing and mass production. In the proximity of a waveguide, a molecule can be coupled to the waveguide mode via evanescent field interaction and the Raman emission may also be collected by the same waveguide. This problem was theoretically modelled by Dhakal et al. in Ref. [54] aiming to optimize the total efficiency of the evanescent excitation and recollection of the spontaneous Raman scattering signal, taking in account the geometry, dimensions and polarization of the waveguide mode and its refractive index difference with the surrounding medium. The authors compared their model with valuable experimental data and numerical simulations of spontaneous Raman emission from Isopropyl Alcohol conducted on photonic waveguides such as slot and strip Silicon Nitride (Si_3_N_4_), Titanium Oxide (TiO_2_) and Silicon on insulator (SOI), i.e. Si waveguide on glass (Fig. 2). It is important to note that the waveguides taken in consideration are purely photonic, i.e. constituted by dielectric materials, and therefore subjected to the law of diffraction.

**Fig. 3:**
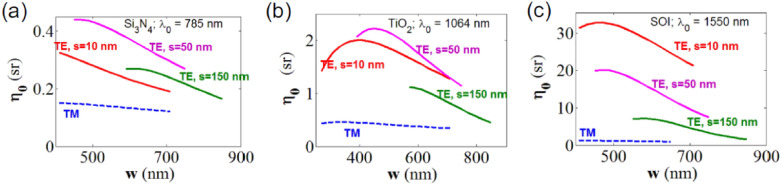
Theoretical conversion efficiency curves for slot waveguides for **a** Si_3_N_4_ core with λ_0_ = 785 nm, **b** TiO_2_ core with λ_0_ = 1064 nm, **c** silicon core with λ_0_ = 1550 nm. The solid lines: TE polarized excitation and collection. Dashed lines: TM polarized excitation and collection. Green, magenta and red lines are respectively for slot width of 150 nm, 50 nm and 10 nm. Only the curve segments corresponding to single mode operation from the cut-off width are shown. (Reprinted with permission from Ref. [54])

The authors showed how, by engineering this system (Fig. 3), it is possible to enhance the overall conversion efficiency (${\eta }_{0}$) compared to the free space case.

The Raman molecule was modelled as a particle with scalar polarizability $\alpha ({\omega }_{p},{\omega }_{s})$ such as the Placzek polarizability [43] for pump and Stokes frequencies ${\omega }_{p}$ and ${\omega }_{s}$, which relates to a scattering cross section $\sigma$ using ${\lambda }_{0}^{4}\sigma ={k}_{\nu }{\alpha }^{2}$, with $2\pi c/{\lambda }_{0}\approx {\omega }_{p}\approx {\omega }_{s}$, and ${k}_{\nu }$ a universal constant related to the coupling between electronic charge and electromagnetic field. In this way it is possible to compute the waveguide scattering efficiency as the ratio of the power that is coupled to the waveguide mode (${P}_{WG}$) and the pumping power (${P}_{pump}$):

3 $$\frac{{P}_{WG}( {{\varvec{r}}}_{0},{\omega }_{s})}{{P}_{pump}}={\Lambda }_{WG}( {{\varvec{r}}}_{0},{\omega }_{p},{\omega }_{s})\sigma ({\omega }_{p},{\omega }_{s})$$

where ${{\varvec{r}}}_{0}$ is the position of the scattering molecule, and

4 $${\Lambda }_{WG}( {{\varvec{r}}}_{0},{\omega }_{p},{\omega }_{s})=\frac{{\pi }^{2}}{{\epsilon }_{0}{k}_{\nu }}\frac{{n}_{g}\left({\omega }_{p}\right){n}_{g}({\omega }_{s})}{n({\omega }_{s})}\frac{{\lambda }_{s}^{2}}{ {\tilde{A }}_{eff}( {{\varvec{r}}}_{0},{\omega }_{p}){\tilde{A }}_{eff}( {{\varvec{r}}}_{0},{\omega }_{s})}$$

is the integrated-luminosity of the waveguide and gives a measure of the fraction of power scattered back to the waveguide. This takes in accounts also the field generated in the surroundings by the waveguide itself, being ${\tilde{A }}_{eff}$ the effective mode area, ${n}_{g},n$ the refractive index respectively of the waveguide and the surrounding.

In the case of an ensemble of molecules with volume density $\rho$ spread over the volume V around the waveguide where the analyte is found, Eq. (3) can be written as a volume integrated contribution as follows:

5 $$\frac{{P}_{WG}( {{\varvec{r}}}_{0},{\omega }_{s})}{{P}_{pump}}=\sigma \left({\omega }_{p},{\omega }_{s}\right) \rho \underset{V}{{\iiint }}{\Lambda }_{WG}\left( {{\varvec{r}}}_{0},{\omega }_{p},{\omega }_{s}\right) d{\varvec{r}}$$

where

6 $${\eta }_{0}\left({\omega }_{p},{\omega }_{s}\right)= \underset{V}{{\iiint }}{\Lambda }_{WG}\left( {{\varvec{r}}}_{0},{\omega }_{p},{\omega }_{s}\right) d{\varvec{r}}$$

is the conversion efficiency. Although the Raman signal can be augmented compared to the free-space case, the enhancement is still far away for what is needed for a competitive platform. In order to increase the enhancement to a level of interest for application, metal structures have to be utilized.

## Slot waveguide assisted enhanced Raman scattering

**Fig. 4:**
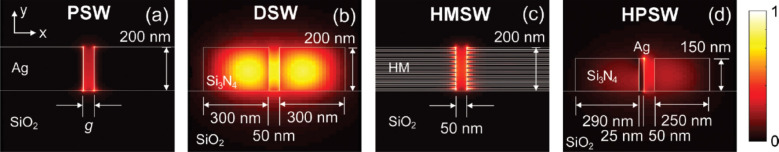
The x–y cross section and EM energy density distribution $w(\rho )\; [\text{J}/\text{m}^{3}]$ of **a** Ag plasmonic slot WG (PSW) with gap width g, **b** Si_3_N_4_ dielectric slot WG (DSW), **c** Ag-Si_3_N_4_ HM plasmonic slot WG (HMSW) with f_m_ = 0.5 (Ag metal layers are shown as gray), and **d** Ag-Si_3_N_4_ hybrid plasmonic slot WG (HPSW). The color bar is in linear scale (normalized units), and each plot is individually normalized. (Reprinted with permission from Ref. [53])

Photonic waveguides are not very well suitable for high on-chip Raman enhancement, given that the enhancement factor is modest and they are diffracted limited. Nevertheless, these platforms could allow a longer light-molecule interactions given the low propagation loss of the photonic mode. In order to obtain a much higher Raman enhancement factor, metallic (i.e. plasmonic) nanostructures and waveguides need be to be employed [55]. Wong et al. [53] have reported a very thorough and complete analysis of various plasmonic waveguides. Additionally, they compared these with a conventional dielectric slot waveguide (DSW), used as a benchmark. The authors have considered a set of different configurations where the fully plasmonic slot waveguide (PSW) can be flanked by hybrid plasmonic-photonic waveguides like a conventional hybrid plasmonic slot waveguide (HPSG) and a multilayer hybrid slot waveguide (HMSW), as depicted in Fig. 4.

They extend the quantum optics approach used in Ref. [56] to the case of waveguides, by utilizing the photon Green function as computed by a semi-analytical normal mode theory [57]. They have also related explicitly the single-molecule enhancement factor (SMEF) of the scattering cross section for a Raman transition, with the spatially averaged Raman enhancement factor (AEF), which highlights the effects of increased light-matter interactions. Furthermore, the authors computed the volume enhancement factor (VEF) to quantify the ability to provide Raman enhancement for an ensemble of molecules. Thanks to this approach they were able to include in the computation the effect of different geometrical parameters, like transversal spacing and length of the slot, and consequently more or less analyte in the active volume of a device. These averaged EFs are specifically defined as a function of the geometrical configuration of a device, where in each specific case a finite-difference time-domain simulation software is used to compute the coupling mode of the electromagnetic field to the waveguide. This methodology allows a meaningfully and realistic comparison of the different configurations in terms of their respective performance (Fig. 4).

**Fig. 5:**
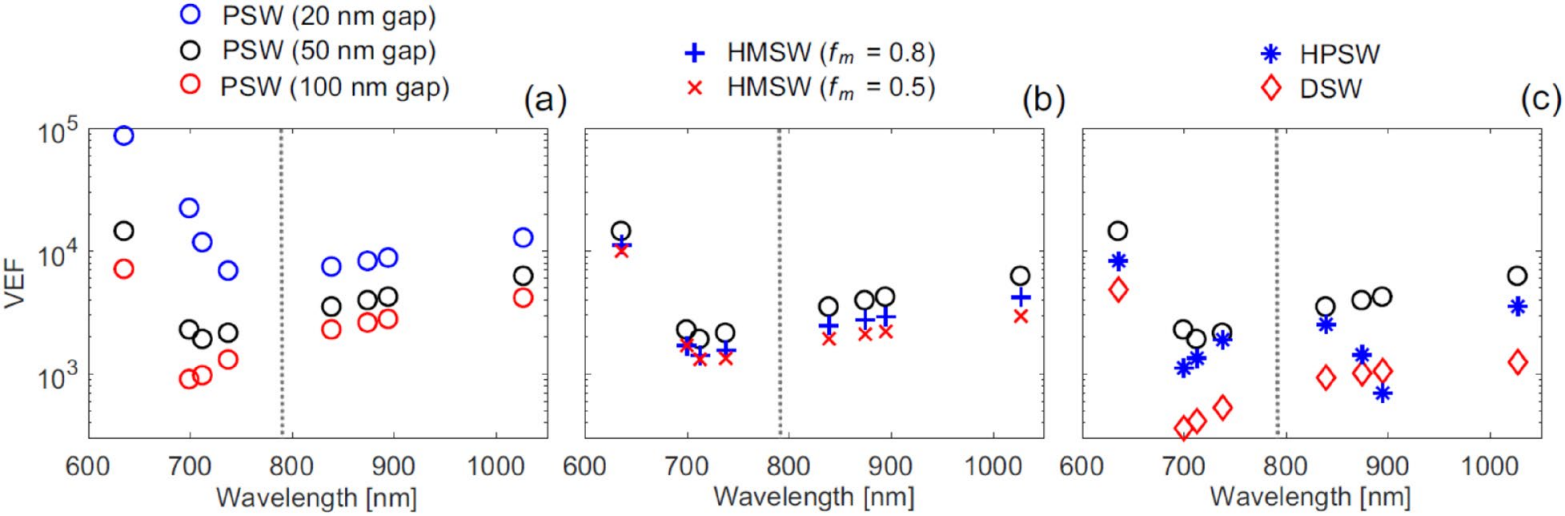
Maximized volume enhancement factor (VEF) with the optimal WG length for the case of in-plane excitation and forward scattered signal collection (see Fig. 1), as a function of wavelength for the Ag plasmonic slot WG (PSW) with gap width g = 50 nm, and other WGs obtained by **a** varying the gap width g: Ag PSW with gap width g = 100 and 20 nm, **b** varying the metal filling fraction f_m_: Ag-Si_3_N_4_ HM slot WG (HMSW) with f_m_ = 0.8 and 0.5, and **c** changing the WG type: Ag-Si_3_N_4_ hybrid plasmonic slot WG (HPSW) and Si_3_N_4_ dielectric slot WG (DSW). The results here are based on comparing to a reference Gaussian beam focused using an objective lens with NA = 0.75 that has a waist diameter D_0_ ≈ 1.28 μm and depth of focus b ≈ 17.4 μm. Note that changing the Gaussian beam parameters will simply scale the VEF. Each marker indicates a specific Stokes or anti-Stokes wavelength corresponding to one of the Raman modes. The vertical dotted line indicates the pump wavelength at λ_0_ = 785 nm. (Reprinted with permission from Ref. [53])

**Fig. 6:**
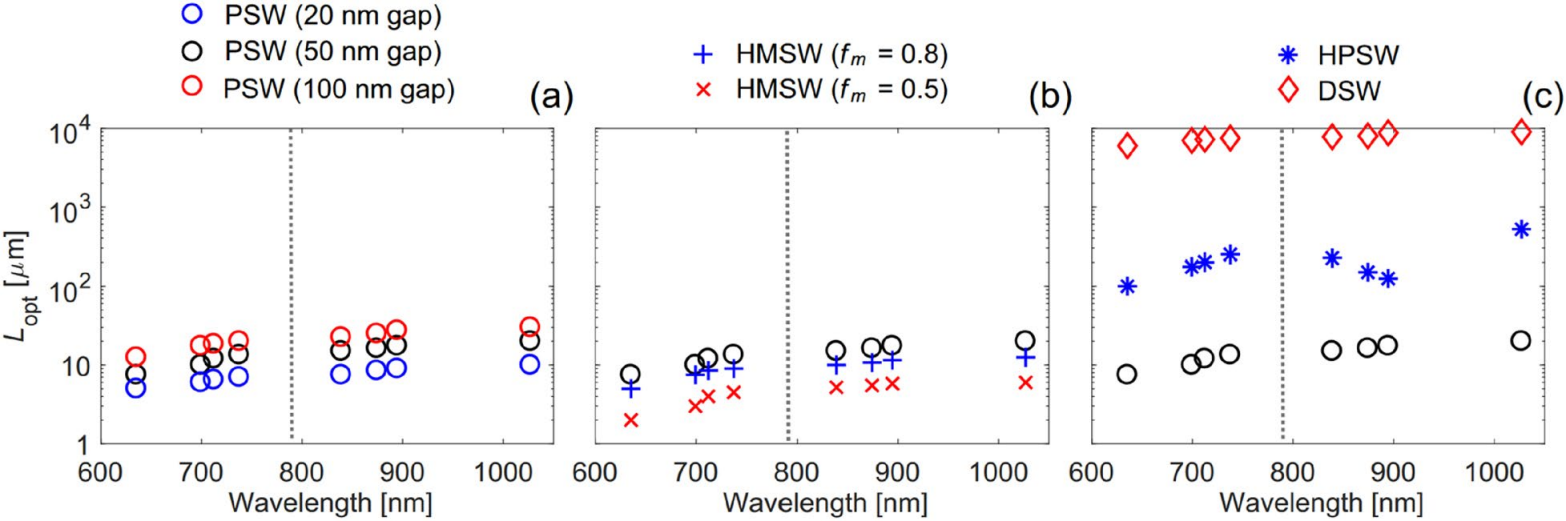
Optimized WG length L_opt_ for maximum spatially averaged Raman enhancement factor (AEF) for the case of forward scattered signal collection, as a function of wavelength for the Ag plasmonic slot WG (PSW) with gap width g = 50 nm, and other WGs obtained by **a** varying the gap width g: Ag PSW with gap width g = 100 and 20 nm, **b** varying the metal filling fraction f_m_: Ag-Si_3_N_4_ HM slot WG (HMSW) with f_m_ = 0.8 and 0 0.5, and **c** changing the WG type: Ag-Si_3_N_4_ hybrid plasmonic slot WG (HPSW) and Si_3_N_4_ dielectric slot WG (DSW). Each marker indicates a specific Stokes or anti-Stokes wavelength corresponding to one of the Raman modes. The vertical dotted line indicates the pump wavelength at λ_0_ = 785 nm. (Reprinted with permission from Ref. [53])

These results underline the best device not only in terms of geometry and materials adopted, but also in terms of performance optimization. Based on this analysis the best VEF is attainable by using the PSW and by reducing the gap (g) of the slot, which doesn’t exhibit any cut-off and therefore it is limited only by fabrication capabilities. However, it is important to realise that smaller gaps imply larger imaginary part of the effective index, i.e. higher Ohmic losses, and therefore a much smaller propagation length as depicted by Fig. 6. By explicitly referring to the Raman transitions of Rhodamine-6G, which is commonly used in literature as a Raman standard, they calculated that for a PSW with a gap width of 20 nm the VEF would climb up to a staggering value of ~ 7373 at scattered wavelength of 839 nm (Fig. 5). This optimized PSW exhibit a total length of 7.5 µm, which shows a VEF about 100 times higher than the one obtained by a conventional Raman spectrometer with hollow core photonic crystal fiber of 75 cm in length.

This study shows that choosing a plasmonic-based on-chip waveguide can in fact be advantageous not only because of its compactness but also because of its enormous enhancement. This is, in general, supplied by the Purcell and electric field enhancement, which are the drivers of the kind of enhancements behind SERS, result to contribute much more than having a larger volume of analyte scattering light.

The plots depicted in Fig. 5 have been calculated at the optimal waveguide length, which implies the following argument: in a plasmonic waveguide system, the higher the field enhancement, i.e. the more compressed the optical field, the shorter the optimal waveguide length is due to the Ohmic loss of the metal. Hence, an optimal length must exist, which is also as a function of the wavelength used. Figure 6 elucidates this concept, where an important design parameter can be extracted: the Purcell and the field enhancements contribute to the Raman scattering enhancement more than the larger light matter-interaction due to augmenting the interaction volume Ref. [53].

**Fig. 7:**
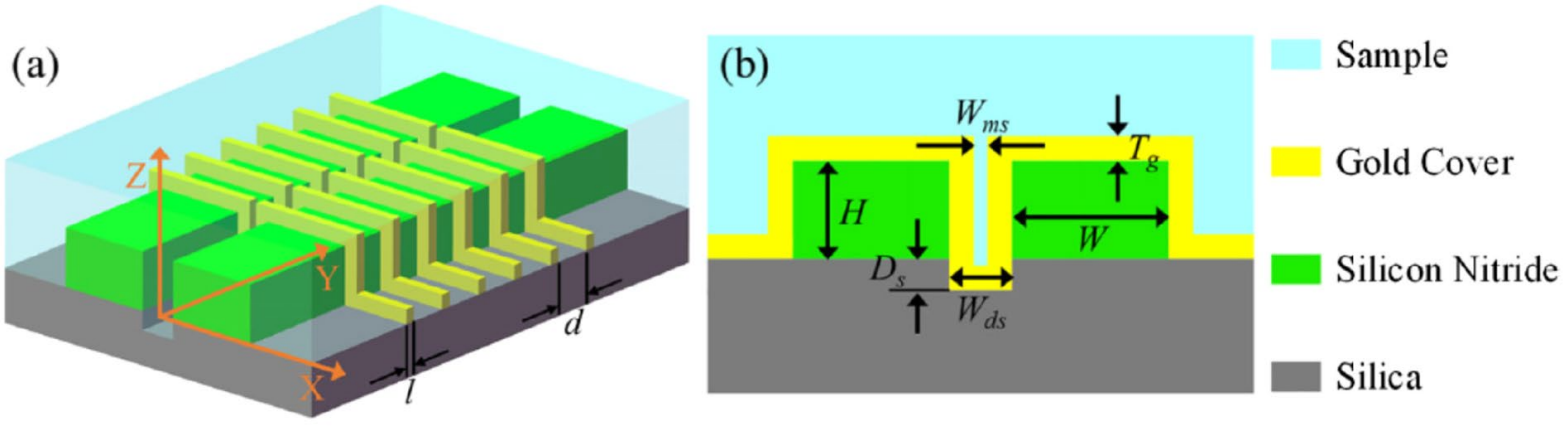
**a** Schematic diagram of the SERS sensor based on the hybrid plasmonic grating slot waveguide. **b** Cross section of the hybrid plasmonic slot waveguide. (Reprinted with permission from Ref. [58])

The Raman scattering of individual molecules in proximity of an optical waveguide can be coupled to the waveguide mode or to free space. This efficiency has been thoroughly estimated, for various slot waveguides, by Wong et al. [53]. It is of note that the coupling efficiency of the Raman scattering signal to the waveguide mode is always at least above 60% regardless the type of photonic, plasmonic or hybrid plasmonic configuration used. Furthermore, a fully plasmonic metal-dielectric-metal slot waveguide (PSW) with 20 nm gap, exhibits a coupling efficiency of more than 97% in the visible (λ > 600 nm) regardless the wavelength used.Following the same reasoning, Li et al. [58] extended theoretically this treatment on a hybrid plasmonic slot waveguide by combining a dielectric slot waveguide with a metallic grating slot structure. Starting from the same idea of the dielectric slot waveguides, that inspired the discussion so far, they show that further enhancement could be obtained by concentrating the electric field on a periodic lattice, by structuring the plasmonic hybrid structures on the dielectric waveguides on separated metal grating (Fig. 7). This last geometry has not yet been studied experimentally and could potentially bring further complications on the side of the fabrication and all the related aspects regarding mass production scaling. Considering that in our opinion, it could not really contribute more than to the order of one percent to the previously discussed VEF, when compared to the best of the devices considered in Ref. [53], it might be actually much more convenient to consider the conventional PSW which can be fabricated easily and potentially manufactured on large scale by using, for instance, techniques like nanoimprinting lithography or atomic layer deposition.

## Experimental comparison of free-space and waveguide-based SERS platforms

The experiments undertaken so far show that the highest values in EF for SERS occurs when the device includes an MDM structure where molecules, usually in an aqueous solution, can be positioned inside the small gap between the two metallic structures. This structure, as theoretically shown in the previous section, induces a very high-volume EF and, suitable for single molecule detection and identification.

For a practical device, SERS spectra have shown to have different empirical dependencies, such as the orientation between a molecule and the polarization of the incident electromagnetic field, points of symmetry breaking given by the alignment of the molecule respect to the metal surface, or the dependency between the dimension of the excitation beam and a useful power density. Due to these variables and the different experimental conditions, it is not straightforward to highlight a common figure of merit that can be used to compare various experimental methods aimed to enhance the Raman Scattering process. There is still not a universal standard by which all the structures tested could be experimentally compared, as, for instance, the average volume EF for each specific device. On the contrary, some authors prefer to use other parameters as figure of merit, such as, for example, the minimum detectable concentration of an analyte of interest.

**Fig. 8:**
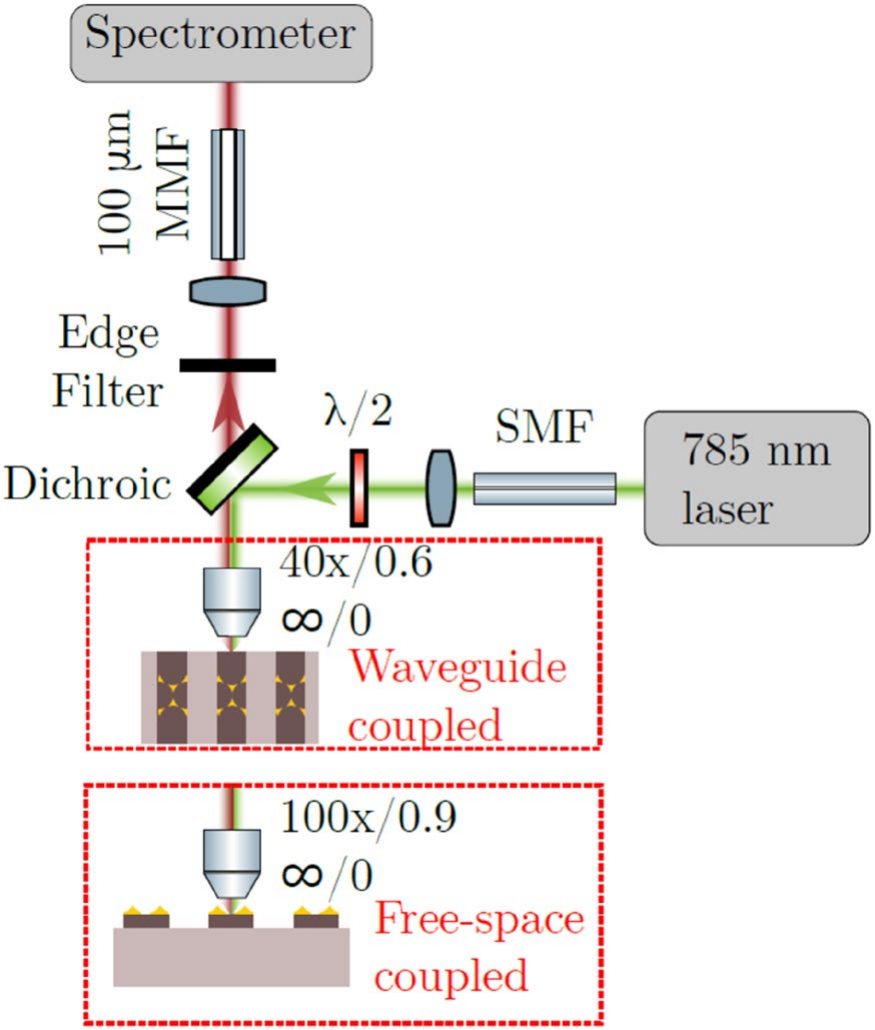
Schematic of the confocal microscope used for collecting Stokes scattered light from both waveguide- and free-space coupled nanotriangles. (Reprinted with permission from Ref. [59])

An appropriate comparison was reported by Wuytens et al. in 2017 [59], where the performance of the nanotriangles fabricated on top of a photonic waveguide was measured by a free space vs a waveguide excitation and detection. In the first case, the patterned surface was aligned perpendicular to the incident pump to implement a free-space excitation scheme. Subsequently, the same device was aligned parallel to the laser source, in the way the excitation was coupled into the silicon nitride waveguide on which the nanotriangles were fabricated. In this case the plasmon resonance excitation was achieved via the evanescent coupling of the waveguide mode (Fig. 8). Figure 8 represent a typical Raman measurement setup for waveguide-based devices. It is evident that both the excitation and the collection can fully happen in free-space rather than through single and multimode fibers as depicted in Fig. 8.

**Fig. 9:**
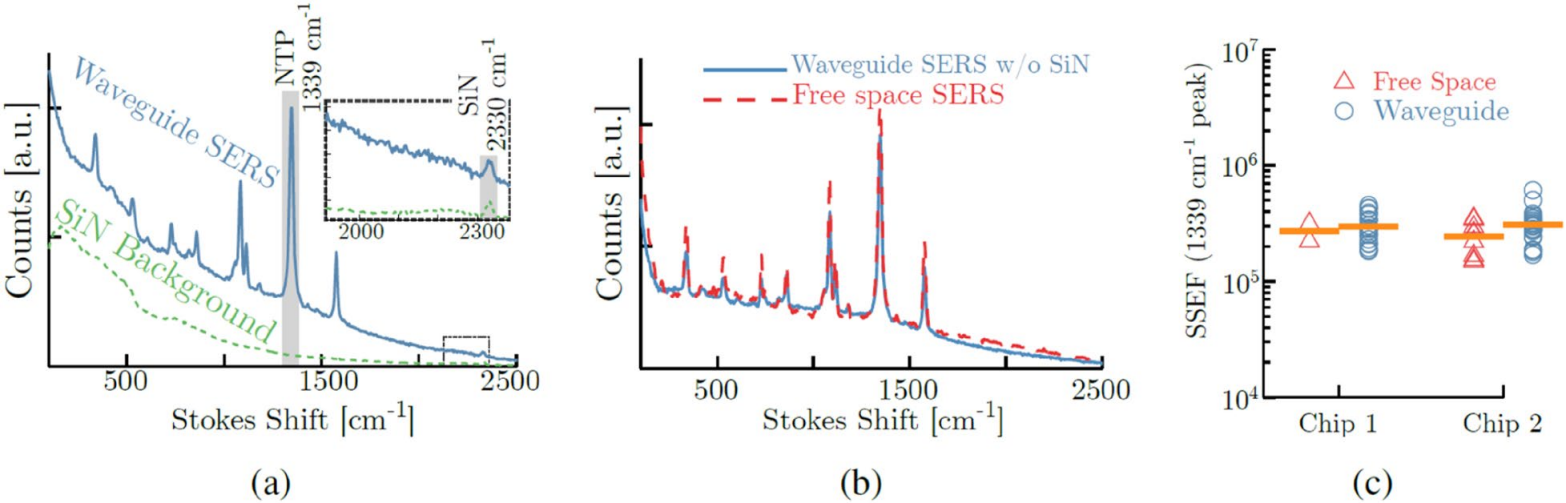
**a** 4-NTP SERS signal acquired through the waveguide (solid blue) and the Si_3_N_4_ background spectrum on a blank reference waveguide (dashed green). The 1339 cm^−1^ is used for quantifying the enhancement factor. The inset shows a characteristic peak for our Si_3_N_4_ at 2330 cm^−1^. **b** Waveguide collected SERS spectrum (solid blue) after subtracting the Si_3_N_4_ background and scaling with the coupling losses, compared to a free-space collected SERS spectrum (dashed red) acquired on the same nanotriangle section. **c** SERS substrate-EF (SSEF) for free-space excitation and collection compared to the signal strength using a waveguide-based measurement, acquired on multiple waveguides on two different chips. (Reprinted with permission from Ref. [59])

The resulting spectra were perfectly overlapping showing an impressive consistency (Fig. 9b). This work shows that the combination of plasmonic antennas with a waveguide excitation and collection is not detrimental compared to the free-space case.

**Fig. 10:**
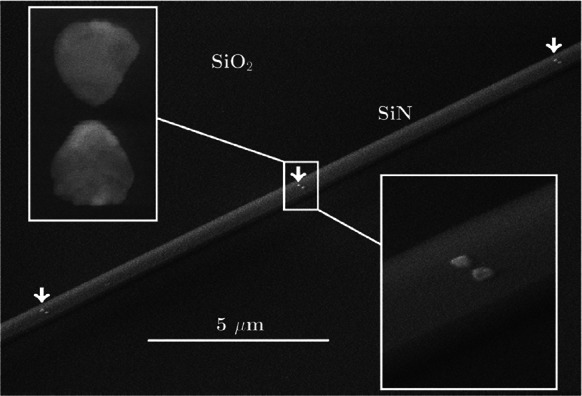
Scanning electron microscope image of a functionalized waveguide. The white arrows indicate antenna positions. The insets show a zoom of a typical antenna. (Reprinted with permission from Ref. [60])

Along the same line, Peyskens et al. [60] experimentally investigated the Raman scattering enhancement obtained by individual plasmonic bowtie antennas fabricated on top of silicon nitride (SiN) waveguides. By measuring recursively Raman spectra, using a series of fabricated waveguides with series of plasmonic nano-bowtie plasmonic antennas positioned on the surface (Fig. 10), they have been able to quantify and model the hybrid function of the enhancement added in comparison to the bare WG.

**Fig. 11:**
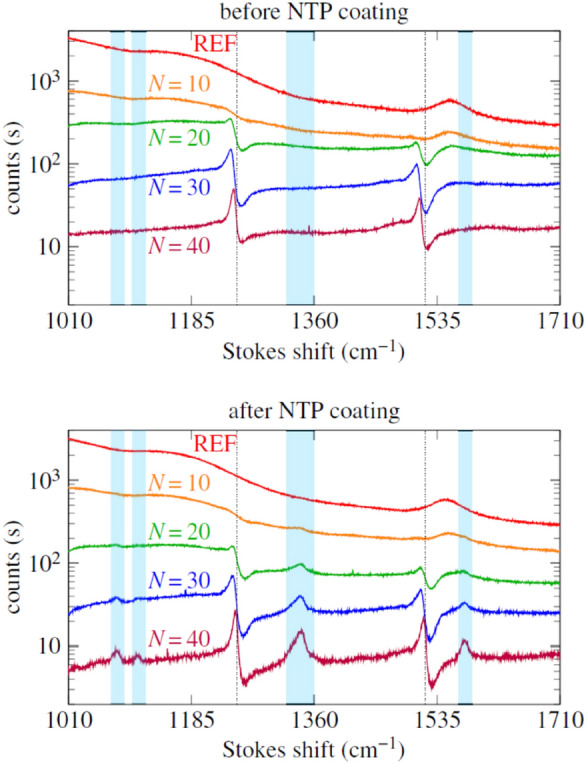
Raman spectra, before and after NTP coating, of a reference waveguide (N = 0) and waveguides functionalized with N = 10, 20, 30, 40 antennas. (Reprinted with permission from Ref. [60])

Here the signal is excited and collected from a SiN waveguide. In a following paper Dhakal A. et al. [61] showed enhanced Raman scattering as a function of the number N of bowtie antennas fabricated onto the SiN waveguide, compared with naked strip and slot waveguides. Finally, from the optimal device, they quantified a Raman conversion efficiency, as defined in Eq. (6), of a film of nitrothiophenol (NTP), equal to ${\eta }_{0}=2.6 \pm 0.77\times {10}^{-15}$ for each single nanobowtie plasmonic antenna (Fig. 11).

The conclusion of their study was that one nanobowtie plasmonic antenna integrated on a silicon nitride waveguide would generate the same Raman signal intensity of 1 μm long bare waveguide. Therefore bare WGs are good enough compared to the integration of plasmonic nanoantennas, which it was shown to provide high enough Raman scattering enhancement.

**Fig. 12:**
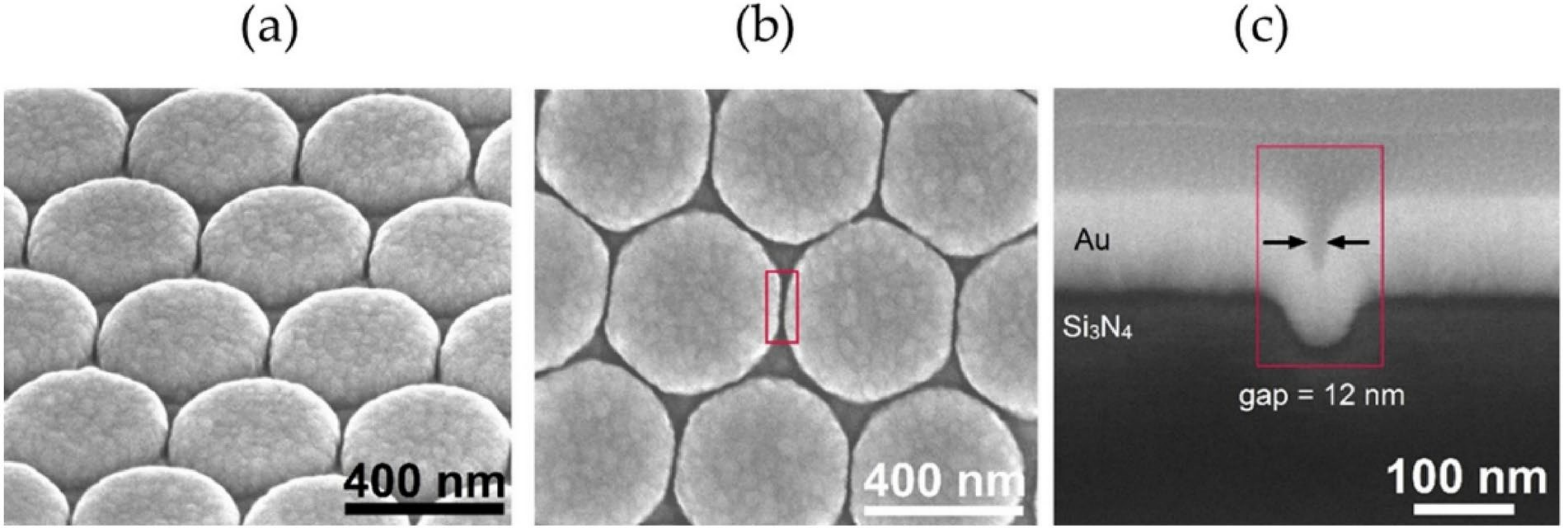
Scanning electron microscope images of gold nanodomes. **a** Tilted view. **b** Top-down view. **c** Cross-section of a nanodome-patterned chip with a 12 nm wide gap between nanodomes. (Reprinted with permission from Ref. [62])

Recently Turk et al. [62] published in 2019 a meaningful comparison, which we find precious for concluding the present discussion about the SERS performance in waveguide assisted devices. They performed SERS measurements on NTP and a peptide obtained by handpicking the top performing enhancement devices, such as the gold nanodomes metasurface [63], in a free-space condition (Fig. 12) and in a plasmonic slot waveguide device. In addition, they included performance results of other waveguide-based SERS devices, such as the integrated bowties [61] and nanotriangles [59] that were previously developed and experimented, to underline the rapid progression in development of such platforms.

**Fig. 13:**
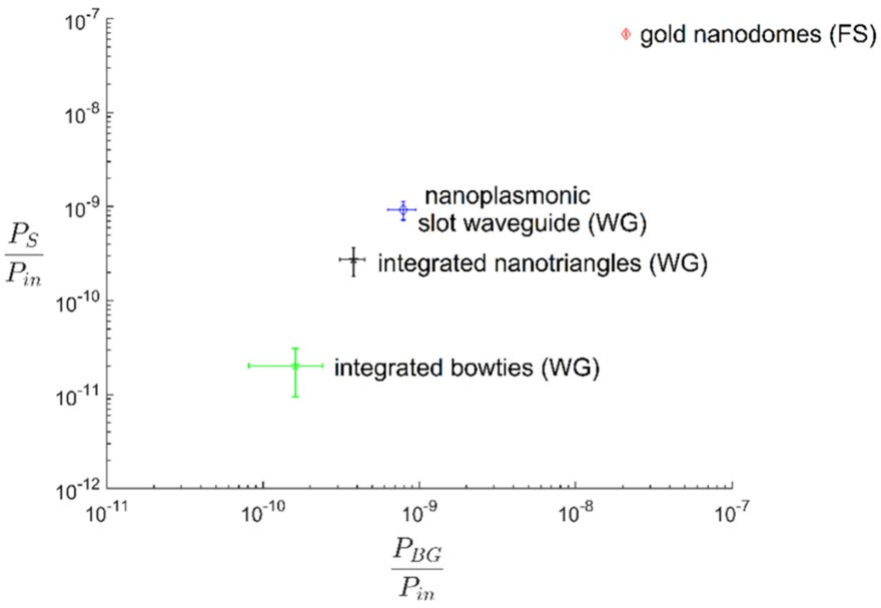
Comparison of SERS background power P_BG_ (x-axis) and SERS Stokes power P_S_ (y-axis) of different SERS platforms. Both parameters are normalized on the input power and the integration time. FS indicates free-space and WG is the waveguide-based excitation. (Reprinted with permission from Ref. [62])

As shown in the concluding graph (Fig. 13), the enhancement for the gold nanodomes in the free-space is the highest, but at the same time it delivers a lot more background signal, which limits the performance of the SERS sensor. The nanoplasmonic slot waveguide instead shows much reduced background signal, while at the same time outperforms other waveguide-based devices, including the integrated bowties.

**Fig. 14:**
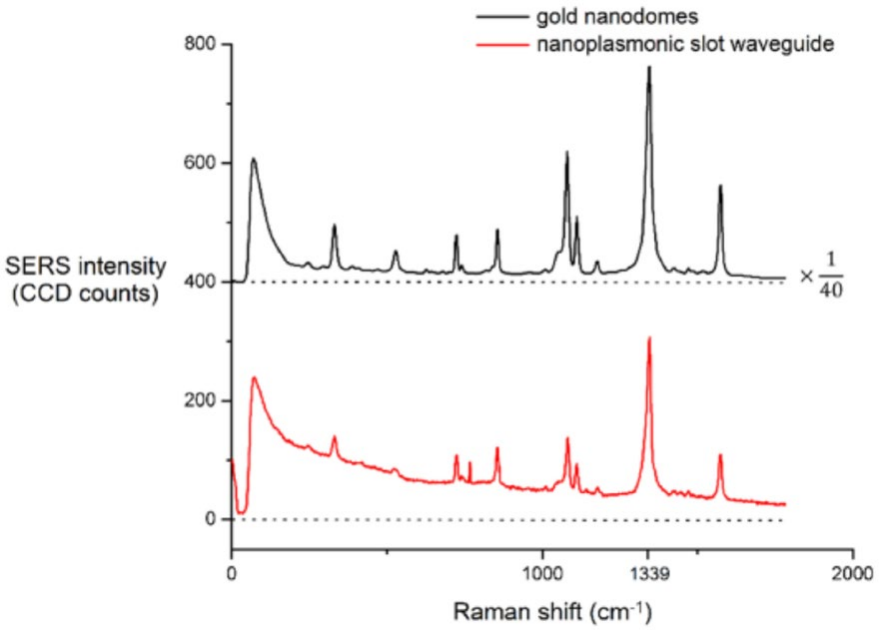
Averaged SERS spectra of the NTP monolayer acquired on gold nanodomes and on the nanoplasmonic slot waveguide. The spectrum on the gold nanodomes was obtained using a laser power of 300 µW and an integration time of 0.13 s. The spectrum on the nanoplasmonic slot waveguide was obtained using a laser power of 350 µW and an integration time of 10 s. The SERS spectrum on the nanodomes was divided by a factor of 40 to allow for better visualization. We subtracted the dark counts, but not the SERS background of the spectra. The spectra are offset on the y-axis for clarity, and the dashed line represents the zero line of each spectrum. Reprinted with permission from Refs. [64]

Following their analysis of the SERS spectra measured from the single layer NTP adsorbate (Fig. 14), the calculated signal-to-background and signal-to-noise ratios (SNRs) remain in favor of the gold nanodomes in free space detection, respectively with a proportion of 3-times and 15-times respect to the nanoplasmonic slot waveguide.

The latter, however, employs a non-resonant SERS enhancement, making the SERS enhancement independent of excited and scattered wavelengths, which offers an advantage when compared to the highly resonant gold nanodomes.

Although the performance of the reported waveguide-based SERS platforms is rapidly improving, hence closing the gap with the free-space devices like the nanodomes, the enormous advantage of an integrated platform like the slot waveguide, resides not only in the fact that the SERS signal can be directly and efficiently collected by the same structure but also in the fact that massive multiplexed measurements can be performed on a large number of analytes, enabling a high-throughput and highly sensitive assays, of particular interest to the pharmacological research and development.

**Fig. 15:**
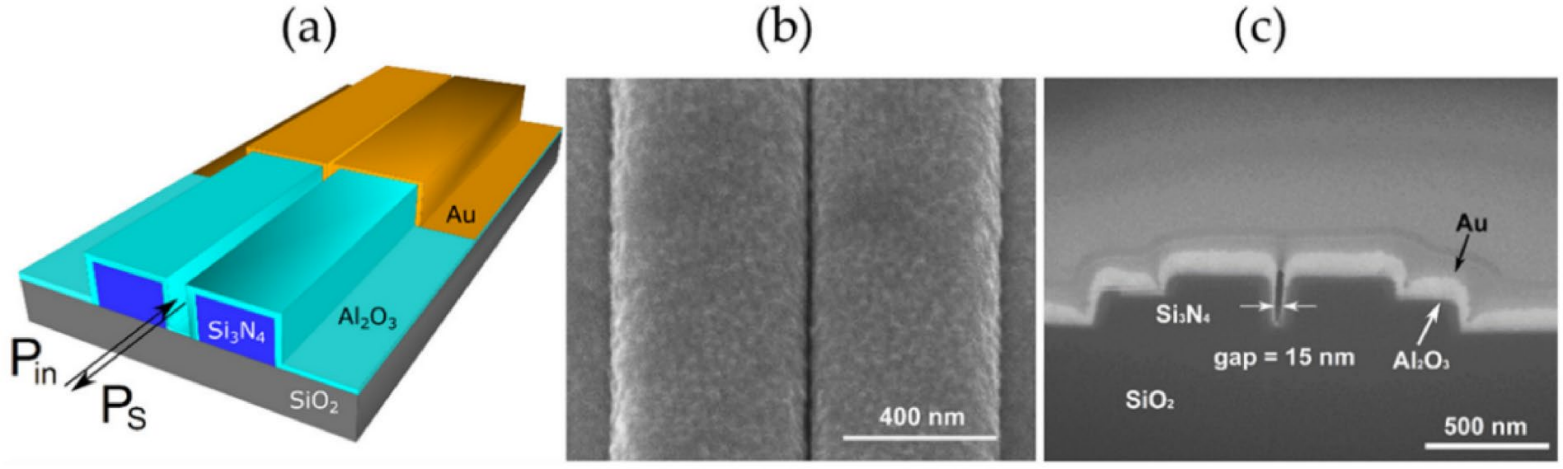
Nanoplasmonic slot waveguide. **a** Schematic showing that the input and Stokes powers are guided by the waveguide. **b** Scanning electron microscope image of the gold-covered slot in top view. **c** Cross-section of a nanoplasmonic slot waveguide with a gap. Reprinted with permission from Ref. [64]

Regarding the slot-waveguide assisted devices, there’re yet other game changing advantages respect to using bulky free-space methods implementing nanoplasmonic antennas for SERS. As insistently remarked in a paper published by Raza et al. [64] in 2018, where other than the discussion about the optimization of the performance of their propagating plasmon assisted slot-WG device, they insist in fact on the difference in the fabrication of those devices (Fig. 15).

The device they obtain has large reproducibility through different devices fabricated (relative standard deviation percentage RSD% < 5%). For the fabrication, they can use plasma enhanced chemical vapor deposition, deep UV lithography and reactive ion-etching for the fabrication of the SiN photonic circuits and atomic layer deposition (ALD) and sputtering for precise metallic coatings, that add the field enhancement and sub-diffractive control needed. They push forward that the fabrication avoids any use of e-beam lithography and that is compatible with the back-end CMOS fabrication, which is a fundamental aspect regarding production and integration in real life devices.

The authors reported a large Raman scattering conversion efficiency ($\sim {10}^{-9}$), thanks not only to the plasmon enhancement of the PSW but also because of the long interaction length of the waveguide, showing a final single molecule enhancement factor $\sim {1.5 \times 10}^{7}$. The authors also demonstrate that such device exhibits a low background, due to the small overlap of the gap plasmon mode with the waveguide mode and a very good Raman scattering spectra reproducibility of the order of less than 5%, collected by various devices. Another very important characteristic is the broadband enhancement that the device exhibits thanks to the gap-plasmon mode, compared, for instance to the localized plasmon enhancement of the bowtie antennas. Such platform is therefore very well suited to future on-chip universal molecular sensors.

**Fig. 16:**
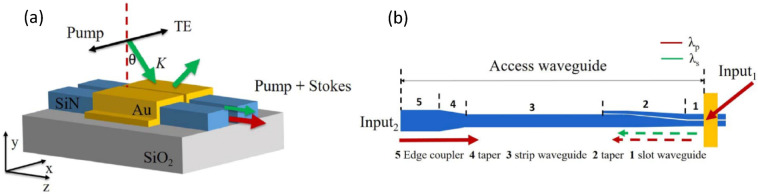
**a** The schematic of plasmonic slot waveguide with oblique illumination in free space. The red dashed line is perpendicular to the top surface of the waveguide. The green and the red arrows indicate the propagation directions of the pump beam and Stokes beam respectively. **b** The Raman plasmonic sensor (yellow) complemented by a Si_3_N_4_ mode converter between slot and strip waveguides, a strip waveguide and a taper for in/out coupling to free space. The red arrows for Input_1_ indicates the out-of-plane pumping beam mode of the excitation, while Input_2_ refers to the pump beam adopted in waveguide coupled mode for the comparison of noise suppression. In both cases the red and green colours refer to pump and Stokes beam respectively. Dashed arrows are the collected light. Adapted with permission from Ref [65], Copyright © 2020, IEEE

A subsequent more recent study published by Li et al. [65] describes the same PSW device but using a different Raman scattering excitation and detection. The latter is collected directly by the waveguide which is excited by an out of plane TE polarized pump forming a specific angle respect to the nanophotonic slot waveguide (Fig. 16).

Their method demonstrates to enhance the signal-to-noise ratio because their device is designed to have a negligible free-space coupling between the pump and the waveguide mode. The coupling efficiency with the gap-plasmon mode can be optimized by varying the incident angle of the pump. To have even more consistent experimental comparison in regard to the SNR improvement due to the reduction of Raman scattering background from the SiN waveguide, they perform equivalent measurements of NTP Raman scattering, using the same sample in two different optical setups, one with oblique free-space excitation and the other via the waveguide mode. In order to accomplish this, the authors added an on-chip a coupler with a taper, coupling free-space light into the strip waveguide mode, which in turn couples into the photonic slot waveguide. The mode is then easily coupled to the gap plasmon of the plasmonic MDM waveguide Fig. 16b.

**Fig. 17:**
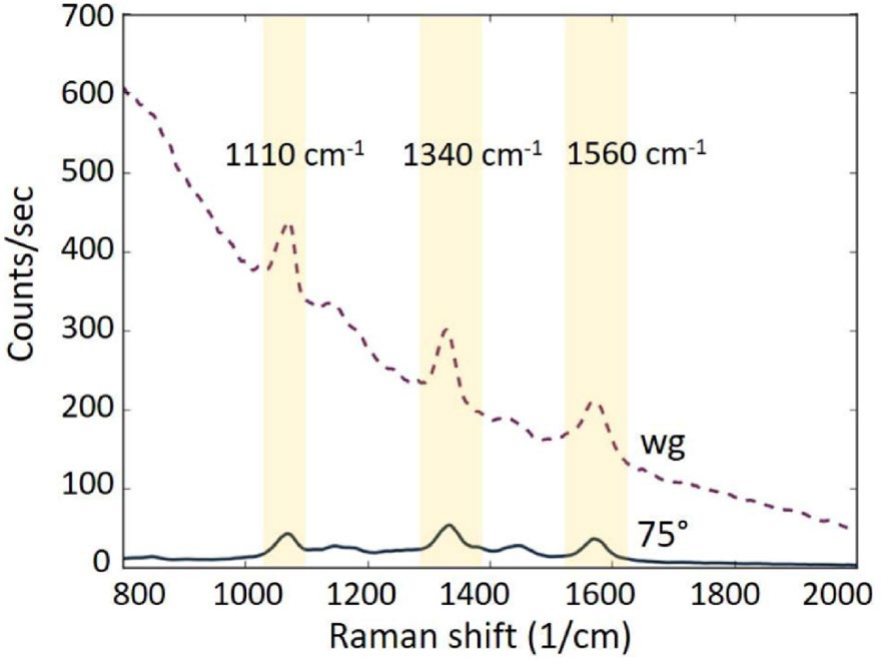
The dashed curve is the measured back-scattered Raman spectrum with the waveguide mode excitation. The solid curve is the spectrum obtained for an oblique free-space excitation at an angle of 75°. The NTP Stokes peaks are highlighted by yellow shaded areas. Adapted with permission from Ref [65], Copyright © 2020, IEEE

The results from the two measurement setups are plotted in Fig. 17 where the effect of background suppression is evidence by using an optimized free-space excitation with an incident angle of 75°. The background signal suppression is approximately of one order of magnitude.

## Waveguide based Raman optical activity

An opportunity for optical analysis of molecules is given by the optical activity (OA) caused by their structural chiral asymmetry. This is particularly relevant for molecules with biological function, as in turn biological functions are inherently sensitive to molecular chirality. The configuration of the multipolar electromagnetic moments depends on the structure of the molecules, which at the same time will cause a difference of cross section in the scattering for electromagnetic waves with different chirality, i.e. circular polarized light. Circular dichroism and optical rotation are very widely used optical measurement techniques for the analysis of chiral materials. In the same way, measurements of Raman optical activity (ROA), which is the result of chiral dependent differential measurements from secondary Raman scattering using different circularly polarized light, are also very valuable to obtain structural information from the vibrational spectrum of the molecules being analyzed, which can allow the determination of the absolute configuration of the molecules [66, 67].

The downside of ROA is that it typically scales further lower in the range of ${10}^{-3}{-}{10}^{-5}$ times when compared to the intensity of classic Raman Scattering spectra. Once again one of the most valued techniques to overcome the loose nature of these phenomena is to use the plasmonic enhancement, to be able to measure surface-enhanced ROA (SEROA) spectra from molecules adsorbed on the metallic surface [68–71].

Nanostructured plasmonic antennas are here the main candidate to be able to take advantage of the sub-diffractive focusing feature of their optical resonances and induce optical chiral field in the near-field to enhance the Raman scattering of close by molecules.

The first result of inducing OA from planar non-chiral metamaterials was from Plum et al. in 2008, where they reported to having used obliquely incident linear polarized light and measured OA from their metamaterials. Another option could be to fabricate 3D plasmonic chiral nanoparticles and use them in an immersion of the analyte of interest and take advantage the enhancement given by the chiral optical near-field to be able to perform SEROA. An interesting idea was theoretically investigated in 2014 by Schäferling et al. [72], considering helical plasmonic nanostructures. They claim that these nano-helices could in fact offer a very promising candidate for the purpose. Potentially a metamaterial fabricated with an array of these objects could be implemented as a SEROA enhancement substrate. However, again fabrication and integration of such devices would not be straightforward.

Some interesting experiments to use plasmonic resonances in coupled silver nanowires have been published by Sun et al. [73] in 2013, where they settled a rather original idea of using the hotspots generated at the proximity coupling of angled silver nanowires to measure hints of the optical activity from an adhesion of chiral FGGO molecules. They remotely excited with polarized light propagating plasmon resonances using the nanowire itself as the WG and were able to control by switching polarization state of the pump light the chirality of the resonance. In this way they reported to having been able to measure a circular intensity difference up to 0.57% at the correspondence of a selected peak of the Raman spectrum, imaging with a microscope complemented with a Raman spectrometer. Although interesting on many aspects, this method seems a good proof of working principle, which however could for example hardly be implemented on mass production devices. In principle they used then achiral plasmonic nanomaterials to obtain in a propagating mode’s interference with a close by nanowire and in the hotspot generated by the interference of the near field of the two objects they were able to enhance and record a sign of ROA.

**Fig. 18:**
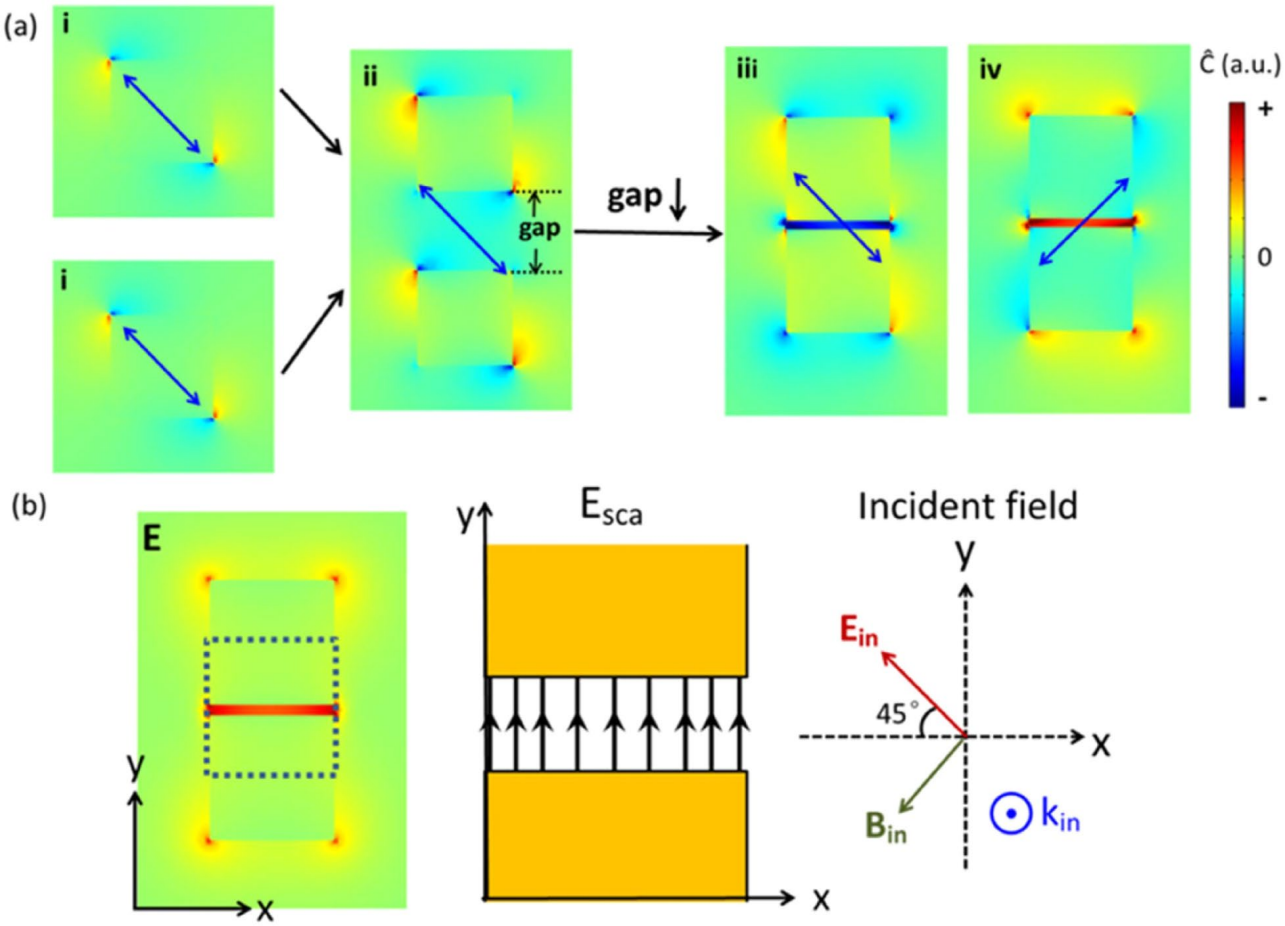
Formation schematic of enhanced chiral near-fields with uniform optical chirality in the gap of a Au block (60 nm × 60 nm × 30 nm) dimer located on glass substrate in water surroundings, excited under polarizations indicated by blue arrows. **a** Chiral near-fields distributions of (i) one Au block, (ii) two blocks with a large gap d of 50 nm, and (iii–iv) block dimers with a small gap d of 5 nm. **b** Corresponding electric fields distribution of the case (a)-iii (left image); the right schematics show directions of incident fields and scattered fields (only fields in the gap are concerned) by a block dimer. All slices are cut from middle positions of the height. (Reprinted with permission from Ref. [75])

In 2012 Schäferling et al. [74] had proposed that the local chiral near-fields that would form when a plasmonic resonance is sustained using oblique linear polarization on a simple square nano-antenna could be used for sensing of chiral molecules. Extending the same idea, in 2015 Tian et al. [75] have instead computed the interference from different geometries of plasmonic dimers and demonstrated using different shapes could be used to create chiral hotspots, induced with linear polarized pumping light. Most interestingly, they have shown how in the nanoscale interstice between rectangular dimers, a very uniform chiral field could be sustained by a plasmonic resonance using an oblique linear polarization respect to their symmetry axis (Fig. 18), which, in our opinion, could well be combined with the PSW design.

## Conclusions

Several converging works have been collected in this paper that could support the possibility of realizing some nanoscale devices based on optical slot-WGs, that functionalized with the near-field enhancement offered by the plasmonic resonance of chosen metals, could allow to be used as commercially viable molecular detectors. To make it simple, could allow to implement a technology of handheld “optical noses”.

Several technologies can converge to obtain an interstitial volume that could be used for an enhancement in the detection of secondary scattered light from any molecule in principle, which could allow to recognize very low concentrations of pollutants, chemicals or allow biological and pharmacological analysis with very limited quantities of analyte.

In particular, PSWs have the potential to be easy enough to fabricate to be scaled up to industrial fabrication, within existing or readily convertible facilities, which is one major advantage respect to other more complex methods that don’t offer inherently such a possibility. In particular, devices that would require free-space detection are presently still more sensitive and still valuable for special purposes, for example in the case of single molecule spectroscopy, but are not suitable for the ready use, miniaturization and integration.

One other advantage of on-chip integrated devices such as for what could be possible for PSWs, is in fact concerning miniaturization and on parallel dedicated array implementation, which could allow an important feature in the practical use that these devices could offer as sensing and measuring instrumentation; this would be particularly advantageous in fields connected to pharmacology or medical diagnostics.

In Sects. 3 and 4 we summarized and connected some publications that could be in our opinion valid references for the working principle of waveguide-based devices for Raman scattering enhancement and detection and represent a reference for the optimization and development of these devices during the very next future.

In Sect. 5 we discussed a comparison with the latest research results delivered regarding the performance and the fabrication of free-space and waveguide-based SERS methods. While still is recognizable that a higher performance in terms of signal-to-background ratios is in favor of the best performing substrates for free-space based excitation and collection of SERS signals, when compared to waveguide-based devices, on the future competition in development and optimization this clearly seems to be a gap that could soon be bridged. Moreover, waveguide-based devices have all the characteristic advantages to be integrated in a full standalone and commercial device, ready to be included in mass production lines.

While in Sect. 6 we discussed some ideas and perspective of future development and application of the same waveguide-based methods, for integrated SEROA that would allow to develop devices that could offer invaluable instruments, particularly regarding biological sciences.

In conclusion, there are still few challenges that the field is currently facing and that need to be solved before any of these platforms become suitable for a commercial device. The most important challenge is to identify the most promising platform that is compact and generates enough enhancement to make the single molecule detection and identification commercially viable. In our opinion the most promising of these reported platforms is the fully plasmonic slot waveguide. This obviously raise another challenge, which resides in the fabrication of the slot with a small gap and its integration with the photonic-based chip which generates the excitation, collects and analyze, on-chip, the Raman scattering signal. Another challenge is given by the fluorescence, which is always an ever-present issue in Raman measurements and needs to be quenched. This can happen with various techniques, e.g. by photobleaching the molecule before preforming a Raman measurement or by bringing it very close to the metal. The latter could potential be an alternative in case the gap of the slot is very small, i.e. few nanometers, which increases the probability to have the molecule closer enough to the metal walls to be quenched during flowing through the channel. Nevertheless, this brings an additional challenge, the molecule adsorption on the metal surface, which obviously must be avoided. We know that this happens on metallic nanostructures by the different molecule–metal chemical affinity. However, in the literature methods to mitigate this issue have been reported, such as a iodine metal modification Ref. [76].

The last challenge that we envision is the integration of such sensor with a micro/nanofluidic system which requires dedicated and potentially complicated fabrication processes as well as nanofluidic configurations and mechanisms to overcome the challenges to couple a very small amount of fluid flowing through the nanochannel sensor.

It is well-known that nanosensors face a diffusion limit challenge when detecting molecules in a very diluted analyte Ref. [77]. There have been solutions to this issue such as localising the nanosensors within super-hydrophobic surfaces that are therefore able to concentrate the molecules where the nanosensor is located Ref. [77]. Other promising solutions involves electro-plasmonic trapping Ref. [78] or nanopores fabricated in a waveguide platform Ref. [79]. Based on these demonstrations, it is our opinion, that the combination of a PSW with a micro/nanofluidic system in a highly multiplexed platform could definitively overcome the unavoidable diffusion limit, allowing detecting and identifying individual molecules even in highly diluted solutions.

However, it is our opinion that these challenges can be overcome in the close future, bringing these platforms to reality into potential applications like universal gas sensors, molecular biomarker sensors which could be potentially implantable (e.g. early cancer sensor, Parkinson, etc.), DNA and genome sequencing for gene theranostics, pollution sensor in water/air systems, airborne small pathogens sensor, etc.

## Data Availability

Not applicable.

## Abbreviations

POC: Point-of-care
LOC: Lab-on-a-chip
RS: Raman spectroscopy
SERS: Surface-enhanced Raman spectroscopy
PR: Plasmonic resonance
EF: Enhancement factor
MDM: Metal-dielectric-metal
SPP: Surface plasmon polariton
ERS: Enhanced Raman scattering
Si_3_N_4_: Silicon nitride
SiN: Silicon nitride
TiO_2_: Titanium oxide
SOI: Silicon on insulator
DSW: Dielectric slot waveguide
HPSG: Hybrid plasmonic slot waveguide
HMSW: Multilayer hybrid slot waveguide
SMEF: Single-molecule enhancement factor
AEF: Averaged Raman enhancement factor
VEF: Volume enhancement factor
PSW: Plasmonic slot waveguide
SSEF: SERS substrate-EF
NTP: Nitrothiophenol
ALD: Atomic layer deposition
SNR: Signal-to-noise ratio
OA: Optical activity
ROA: Raman optical activity
SEROS: Surface-enhanced ROA
